# Use of machine learning models to predict in‐hospital mortality in patients with acute coronary syndrome

**DOI:** 10.1002/clc.23957

**Published:** 2022-12-07

**Authors:** Rong Li, Lan Shen, Wenyan Ma, Bo Yan, Wenchang Chen, Jie Zhu, Linfeng Li, Junyi Yuan, Changqing Pan

**Affiliations:** ^1^ Clinical Research Center, Shanghai Chest Hospital Shanghai Jiao Tong University Shanghai China; ^2^ Yidu Cloud Technology Inc. Beijing China; ^3^ Information Center, Shanghai Chest Hospital Shanghai Jiao Tong University Shanghai China; ^4^ Hospital's Office, Shanghai Chest Hospital Shanghai Jiao Tong University Shanghai China

**Keywords:** acute coronary syndrome, in‐hospital mortality, logistic regression, machine learning, XGBoost

## Abstract

**Background:**

Cardiovascular diseases are a significant health burden with the prevalence increasing worldwide. Thus, a highly accurate assessment and prediction of death risk are crucial to meet the clinical demand. This study sought to develop and validate a model to predict in‐hospital mortality among patients with the acute coronary syndrome (ACS) using nonlinear algorithms.

**Methods:**

A total of 2414 ACS patients were enrolled in this study. All samples were divided into five groups for cross‐validation. The logistic regression (LR) model and XGboost model were applied to predict in‐hospital mortality. The results of two models were compared between the variable set by the global registry of acute coronary events (GRACE) score and the selected variable set.

**Results:**

The in‐hospital mortality rate was 3.5% in the dataset. Model performance on the selected variable set was better than that on GRACE variables: a 3% increase in area under the receiver operating characteristic (ROC) curve (AUC) for LR and 1.3% for XGBoost. The AUC of XGBoost is 0.913 (95% confidence interval [CI]: 0.910–0.916), demonstrating a better discrimination ability than LR (AUC = 0.904, 95% CI: 0.902–0.905) on the selected variable set. Almost perfect calibration was found in XGBoost (slope of predicted to observed events, 1.08; intercept, −0.103; *p* < .001).

**Conclusions:**

XGboost modeling, an advanced machine learning algorithm, identifies new variables and provides high accuracy for the prediction of in‐hospital mortality in ACS patients.

## 1 INTRODUCTION

Acute coronary syndrome (ACS), including unstable angina (UA), ST‐segment elevation myocardial infarction (STEMI), and non‐ST‐elevation myocardial infarction (NSTEMI), represent a life‐threatening manifestation of atherosclerotic progression in the coronary territory. Due to the improvement of surgical technology and emergency corresponding measures in the field of ACS, in‐hospital mortality has decreased over the past few decades. However, there still be a stubbornly high in‐hospital mortality caused by ACS each year, suggesting that more effective precautions are needed.

Although many risk models for predicting in‐hospital mortality had been developed for patients with ACS,^1^, ^2^, ^3^, ^4^, ^5^, ^6^, ^7^, ^8^, ^9^ the most typical one of which is the global registry of acute coronary events (GRACE),^2^ there are several major limitations of these existing models. First, some classic scores^2^, ^3^, ^4^ were developed nearly 2 decades ago, and may not be suitable for current practice with advances in treatment and awareness of risk management. Second, some studies^3^, ^5^, ^6^ were designed for risk assessment early after the patient presentation, and some important variables, such as left ventricular function, coronary angiography, cardiac biomarkers, and ST‐segment resolution were not included. Third, some studies had revealed the relationship between laboratory markers or vital signs at admission and in‐hospital mortality.^10^, ^11^, ^12^, ^13^, ^14^, ^15^, ^16^, ^17^ However, dynamic changes in markers such as cardiac troponin I (cTnI) are not included in these models. Finally, compared to existing risk models using linear algorithms such as logistic regression (LR), machine learning techniques, such as extreme gradient boosting (XGBoost), have been widely applied in predicting clinical events in virtue of their capabilities for modeling complex interactions among variables^18^ and nonlinear relationship between variables and events.

Therefore, expanding existing risk models with dynamic changes of markers and application of machine learning (ML) techniques would offer potential room for improvement in the accuracy of predicting in‐hospital mortality in patients with ACS. The present study aims to develop and validate predictive models from a retrospective cohort collected from electronic health records. The retrospective cohort includes 2414 patients diagnosed with ACS admitted to Shanghai Chest Hospital in Shanghai, China, from 2015 to 2021.

## 2 MATERIALS AND METHODS

### 2.1 Study population

This retrospective cohort study took place at Shanghai Chest Hospital. Patients with ACS presenting with UA, STEMI, or NSTEMI were eligible for inclusion in this study. ACS was defined according to the current guidelines.^19^ Patients who were younger than 18 were excluded. Patients who died within 24 h of admission were excluded. After the exclusion, 2414 hospitalized patients were analyzed in this study. This study was approved by the Ethics Committee of Shanghai Chest Hospital with approval number KS(P)22005. Since the data were collected respectively, consent was not required.

### 2.2 Data collection

A total of 67 variables were collected from electronic medical records based on previous studies and clinical experience. Variables include patients' demographics, medical and personal history, presentation information, electrocardiographic characteristics, echocardiography characteristics, surgical characteristics, and initial laboratory values. In addition, we included the changes in repeated measured variables which reflected variations in patients' status during hospitalization. The repeated‐measured variables include heart rate (HR), systolic blood pressure (SBP), diastolic blood pressure (DBP), creatinine (Cre), cTnI, myoglobin (MB), creatine kinase‐MB (CK‐MB), and brain natriuretic peptide (BNP). To investigate the relationship between such variables and outcome, first, we collected information on whether one repeated measured variable elevated significantly after admission. Elevation of HR, SBP, or DBP was defined as the difference of more than 20 units between the maximum value and the first one during the hospitalization. Elevation of Cre, cTnI, MB, CK‐MB, or BNP was defined as the difference of more than one standard deviation (SD) of the mean value between the maximum value and the first one during the hospitalization. Second, we calculated the value of variable change before the end event, discharging, or death. The change of the repeated variables mentioned above was defined as the difference between the last value and the second to last value during the hospitalization. If only one measurement exists, the change is defined as zero. The outcome of interest in this study was all‐cause mortality during hospitalization.

### 2.3 Data preprocessing

We developed models using two algorithms: LR and extreme gradient boosting (XGBoost). We applied different data preprocessing steps to different models according to their characteristics to achieve the best performance. For the LR model, we discretized laboratory variables to avoid the influence of extreme values. Log transformation was applied before the discretization if there was too much skewness in the variable. The discretization approach was to calculate how many SDs the value was away from the mean value. Separation points are 3, 2, −2, and −3. Values that are three SDs larger than the mean would get the largest new value of 4. Values 2–3 SDs would get 3, and −2 to 2 SDs (usually the reference range) would get 2. Following this discretization method, laboratory values were transformed into a range of 0–4. In addition, we conducted normalization of all variables since we used L2 regulation for LR. We tried both preprocessing steps mentioned above and no preprocessing step for XGBoost and chose the model with better performance.

### 2.4 Feature selection

In this study, we used two sets of variables for model construction. Since the current standard model for ACS in‐hospital mortality prediction was the GRACE using eight variables, we use these eight variables to derive the baseline model. The process of feature selection is divided into two steps. First, we expanded the variable set which was chosen after univariate LR analysis (Supporting Information: Table S1). Only variables with a *p* < .05 in the univariate analysis were entered into the candidate variable set. Secondly, correlation analysis was performed. If two variables were highly correlated (|PCC| > 0.6, the absolute value of Pearson correlation coefficient), only one was selected (Supporting Information: Table S2 and Figure S1) as the candidate variable. After the two steps of variable selection, several variables were retrieved into the set based on clinical considerations, including the history of heart failure, history of myocardial infarction, history of percutaneous coronary intervention (PCI), presentation in cardiac arrest, ST‐segment elevation, CK‐MB, MB, and BNP (Supporting Information: Table S3). To impute missing values in variables (Supporting Information: Table S4), we used random forest regression^20^ which derives predicted values for the missing values. The prediction was based on variables without missing values.

### 2.5 Modeling construction

All samples were randomly divided into five groups for cross‐validation (CV). Splitting was performed before any preprocessing steps. In each subset, the total number of samples and deaths were almost the same. During each learning, four subsets were used for training and the left one was used for validation. Thus, each derivation cohort had about 80% samples (*n* = 1932) and each validation cohort had about 20% samples (*n* = 482). We compared two methods: LR and XGBoost. LR is a very traditional and widely used model owing to its advantages in efficiency and interpretability. For LR models, we conducted L2 regulation to achieve better model performance. XGBoost is an efficient system implementation of gradient boosting that makes predictions by evaluating and aggregating the results of multiple decision trees. XGBoost is able to fit nonlinear relationships that exist between variables and the outcome. A nested k‐fold CV‐based grid search was used for hyperparameter selections. We used all candidate variables to build the initial XGBoost model. For ease of application, feature subsets with top 5, top 10, and top 20 variables were selected according to the ranked importance of the variables.

### 2.6 Statistical analysis

Data were presented as the mean ± SD for normally distributed data, or medians and interquartile range for non‐normally distributed data. Normally distributed variables were compared using Student's *t*‐test and non‐normally distributed variables were compared using the Mann–Whitney *U* test. Categorical data were expressed as numbers and percentages (%). Pearson's *χ* ^2^ test or Fisher's exact test were used for categorical data, as appropriate. Model discrimination was assessed using the area under the receiver operating characteristic (ROC) curve (AUC). DeLong's test was used to compare the ROC curves. In addition, we calculated the sensitivity (recall), *F* score, specificity, positive predictive value (precision), and negative predictive value.

Model calibration was tested by (1) calculating the slope and intercept of the calibration curve and (2) the Brier score. For perfect calibration, the calibration slope equals 1 and the intercept equals 0. And the smaller the Brier score is, the better calibration will be. Shapley additive explanations (SHAP) were used to evaluate the importance of variables.^21^

All analyses were conducted in open‐source Python, version 3.7.9 (Python Software Foundation), and R software, version 4.0.3 (R Foundation for Statistical Computing).

## 3 RESULTS

### 3.1 Population characteristics

A total of 2414 patients (mean [SD] age, 66.4 [11.6] years; 1817 [75.3%] male) were enrolled in our study. There were 85 in‐hospital deaths with a rate of 3.5% in the entire cohort. The median length of hospital stay was 5.1 days. The median time of death was 5.0 days after hospital presentation. There is no significant difference between the length of hospital stay of alive and dead patients. About 20% of deaths occurred within 24 h of hospital admission. Table 1 includes characteristics of the surviving and dead patients. A total of 810 patients (33.6%) had diabetes, 1613 (66.8%) had hypertension, and 259 (10.7%) had experienced a prior cerebral stroke. In addition, 54 patients (2.2%) had experienced CABG surgeries and 833 (34.5) had experienced PCI surgeries.

**Table 1:** Baseline characteristics of patients

Characteristic	Survived (*n* = 2329)	Died (*n* = 85)	*p* Value
Demographic characteristic			
Male sex	1762 (75.7)	55 (64.7)	.022
Age, year	66 (59, 74)	74 (67, 83)	<.001
BMI, kg/m^2^	24.2 (22.4, 26.4)	22.3 (19.8, 24.8)	<.001
Marital status			.281
Married	2319 (99.6)	84 (98.8)	
Unmarried	6 (0.3)	1 (1.2)	
Other	4 (0.2)	0 (0)	
Type of Insurance			.003
Type 1^a^	71 (3)	2 (2.4)	
Type 2^a^	1457 (62.6)	70 (82.4)	
No insurance	799 (34.3)	13 (15.3)	
Other	2 (0.1)	0 (0)	
Medical and personal history			
DM	768 (33)	42 (49.4)	.002
HTN	1559 (66.9)	54 (63.5)	.512
CVA	247 (10.6)	12 (14.1)	.304
HF	170 (7.3)	8 (9.4)	.464
MI	37 (1.6)	2 (2.4)	.583
PAD	175 (7.5)	9 (10.6)	.294
COPD	34 (1.5)	4 (4.7)	.018
HLP	172 (7.4)	4 (4.7)	.351
Renal dysfunction	191 (8.2)	31 (36.5)	<.001
CABG	52 (2.2)	2 (2.4)	.941
PCI	808 (34.7)	25 (29.4)	.314
Family history of CHD	53 (2.3)	0 (0)	.315
Smoking	551 (23.7)	14 (16.5)	.184
Drinking	160 (6.9)	4 (4.7)	.412
Presentation			
HR, beats/min	74 (69, 82)	96 (79.5, 103.5)	<.001
SBP, mmHg	132 (120, 148)	120 (103, 136.8)	<.001
DBP, mmHg	78 (70, 85)	70 (60, 80)	<.001
In CHF	147 (6.3)	27 (31.8)	<.001
In CS	10 (0.4)	10 (11.8)	<.001
After CA	3 (0.1)	1 (1.2)	.02
Killip			<.001
I	2210 (94.9)	35 (62.4)	
II	81 (3.5)	14 (16.5)	
III	14 (0.6)	2 (2.4)	
IV	24 (1)	16 (18.8)	
NYHA			<.001
I	1489 (63.9)	50 (58.8)	
II	420 (18.0)	5 (5.9)	
III	330 (14.2)	13 (15.3)	
IV	90 (3.9)	17 (20)	
UCG findings			
LVEF, %	61 (55, 64)	45 (34.2, 55.8)	<.001
LVESD, mm	31 (28, 35)	36 (30, 46)	<.001
LVEDD, mm	48 (45, 52)	50 (43.5, 59)	.089
LAD, mm	37 (35, 40)	40.5 (36.2, 44)	<.001
MR			<.001
No	564 (24.2)	8 (9.4)	
Mild	1170 (50.2)	28 (32.9)	
Moderate	260 (11.2)	21 (24.7)	
Severe	16 (0.7)	4 (4.7)	
Unknown	319 (13.7)	24 (28.2)	
ECG findings			
ST‐segment elevation	676 (29)	42 (49.4)	<.001
ST‐segment depression	220 (9.4)	15 (17.6)	.012
ST‐segment deviation	1021 (43.8)	50 (58.8)	.006
T wave Inverted	322 (13.8)	7 (8.2)	.14
ST‐T deviation	1844 (79.2)	64 (75.3)	.388
CLBBB	34 (1.5)	6 (7.1)	<.001
CRBBB	141 (6.1)	16 (18.8)	<.001
Initial laboratory values			
Cre, µmol/L	75 (63,90)	100.5 (79.8, 128)	<.001
cTnI, ng/ml	0.2 (0, 4.1)	3.6 (0.7, 60.9)	<.001
MB, ng/ml	34.2 (21.2, 84.5)	151 (53.1, 428.4)	<.001
CK‐MB, ng/ml	3 (1.4, 19.4)	8.3 (3.2, 73.6)	<.001
BNP, pg/ml	98 (36, 281)	534 (224.5, 1274.2)	<.001
In‐hospital status variation			
Elevated HR	390 (16.7)	24 (28.2)	.006
Elevated SBP	246 (10.6)	11 (12.9)	.485
Elevated DBP	118 (5.1)	9 (10.6)	.025
Elevated Cre	312 (13.4)	36 (42.4)	<.001
Elevated cTnI	838 (36)	25 (29.4)	.214
Elevated MB	332 (14.3)	29 (34.1)	<.001
Elevated CK‐MB	487 (20.9)	20 (23.5)	.56
Elevated BNP	304 (13.1)	19 (22.4)	.013
HR change, beats/min	0 (−6, 6)	1 (−4.5, 17)	.017
DBP change, mmHg	0 (−8, 5)	−6 (−9, 8.2)	.725
SBP change, mmHg	−3 (−13, 7)	4 (−2.5, 10.2)	.06
Cre change, µmol/L	1 (−4, 6)	37 (−6, 102)	<.001
cTnI change, ng/ml	−0.1 (−1.5, 0.1)	0 (−1.8, 1.2)	.101
MB change, ng/ml	−3.2 (−15.9, 1.8)	50.7 (−14, 464.8)	<.001
CK‐MB change, ng/ml	−0.3 (−2.8, 0.6)	−0.2 (−5.7, 1.6)	.831
BNP change, pg/ml	40.5 (−8, 109.8)	53 (−76.8, 600.5)	.656
In‐hospital surgical characteristics			
CABG	209 (9)	5 (5.9)	.325
PCI	1585 (68.1)	39 (45.9)	<.001
LM stenosis, %	70 (50, 85)	80 (70, 91.2)	.053
LAD* stenosis, %	90 (80, 99)	99.5 (90, 100)	<.001
LCX stenosis, %	85 (70, 95)	95 (88.8, 100)	.017
RCA stenosis, %	90 (70, 100)	95 (86.2, 100)	.026

Abbreviations: BMI, body mass index; BNP, brain natriuretic peptide; CA, cardiac arrest; CABG, coronary artery bypass graft; CHD, coronary heart disease; CHF, congestive heart failure; CK‐MB, creatine kinase‐MB; CLBBB, complete left bundle branch block; COPD, chronic obstructive pulmonary disease; CRBBB, complete right bundle branch block; Cre, creatinine; cTnI, cardiac troponin I; CVA, cerebral stroke; CS, cardiogenic shock; DBP, diastolic blood pressure; DM, diabetes mellitus; ECG, electrocardiography; HF, heart failure; HLP, hyperlipidemia; HR, heart rate; HTN, hypertension; LAD*, left anterior descending; LAD, left atrial diameter; LCX, left circumflex; LM, left main; LVEDD, left ventricular end‐diastolic diameter; LVEF, left ventricular ejection fraction; LVESD, left ventricular end‐systolic diameter; MB, myoglobin; MI, myocardial infarction; MR, mitral regurgitation; NYHA, New York Heart Association;PAD, peripheral artery disease; PCI, percutaneous coronary intervention; RCA, right coronary artery; SBP, systolic blood pressure; UCG, echocardiography.

^a^ Type 1: Nonemployed resident medical insurance and Type 2: urban employee medical insurance.

### 3.2 Model construction and evaluation

**Figure 1:**
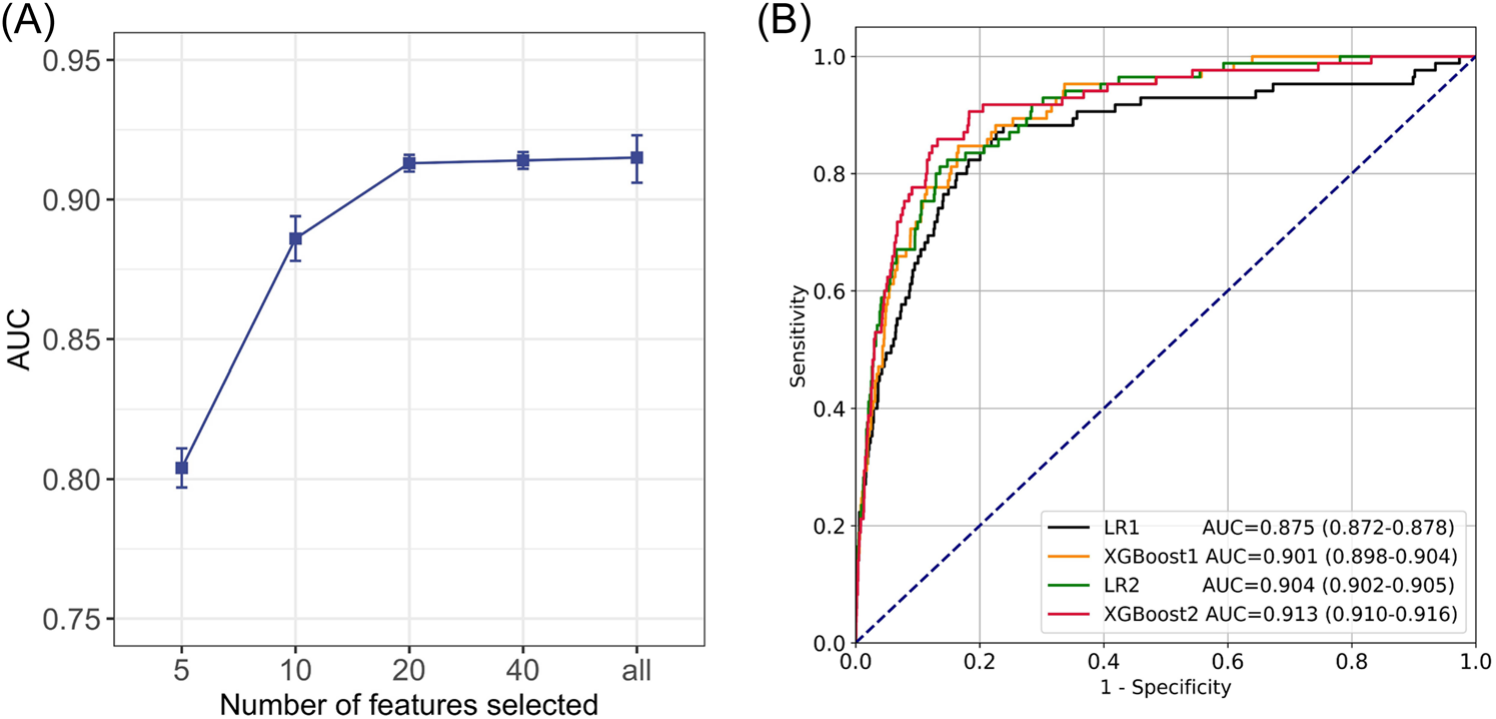
AUC of the XGBoost model with the different number of features and receiver operating characteristic curves of four models on the selected variable set. (A) AUC of the XGBoost model with a different number of features. (B) Receiver operating characteristic (ROC) curves of four models on the selected variable set. *Note*: LR1: Logistic regression model with the same variables of GRACE. XGBoost1: XGBoost model with the same variables of GRACE. LR2: Logistic regression model with selected variables. XGBoost2: XGBoost model with selected variables. AUC, area under the ROC curve.

The AUCs of XGBoost models constructed with top 5, top 10, and top 20, and all candidate features were 0.804 (95% confidence interval [CI]: 0.797–0.811), 0.886 (95% CI: 0.878–0.894), 0.913 (95% CI: 0.910–0.916), and 0.914 (95% CI: 0.911–0.917), respectively (Figure 1A). When the number of variables reached 20, the model performance did not improve with the increase in the number of variables. These 20 variables were used as selected to construct the final model. The variables of GRACE, candidate and selected were shown in Supporting Information: Table S3. In models which used original GRACE variables, XGBoost had improvements in discrimination over LR using the same data inputs (Table 2). The Delong test showed a significant difference (*p* = .01). The AUC of the XGBoost model with selected variables was higher than the LR model, with corresponding improvements in sensitivity and specificity. The XGBoost model achieved an AUC of 0.913 compared with 0.904 of the LR model. As a result, the XGBoost model derived from the selected variable set has the highest AUC and best performance compared with GRACE and the XGBoost model with the same variables of GRACE (*p* < .001, Delong test) (Figure 1B).

**Table 2:** Performance characteristics of models for predicting in‐hospital mortality in ACS

	GRACE		Selected variables	
	Logistic regression	XGBoost	Logistic regression	XGBoost
ACC (95% CI)	0.835 (0.833–0.837)	0.838 (0.834–0.842)	0.831 (0.829–0.833)	0.857 (0.853–0.862)
AUC	0.875 (0.872–0.878)	0.901 (0.898–0.904)	0.904 (0.902–0.905)	0.913 (0.910–0.916)
Sensitivity	0.821 (0.811–0.831)	0.824 (0.814–0.834)	0.841 (0.829–0.853)	0.845 (0.829–0.860)
Specificity	0.834 (0.832–0.836)	0.838 (0.835–0.841)	0.831 (0.829–0.853)	0.858 (0.853–0.862)
PPV	0.154 (0.152–0.156)	0.157 (0.155–0.159)	0.154 (0.151–0.156)	0.178 (0.172–0.184)
NPV	0.992 (0.992–0.993)	0.992 (0.992–0.993)	0.993 (0.993–0.994)	0.993 (0.993–0.994)
*F* score	0.260 (0.257–0.263)	0.263 (0.259–0.267)	0.263 (0.256–0.263)	0.294 (0.285–0.304)
Brier score	0.165 (0.163–0.167)	0.162 (0.159–0.166)	0.169 (0.167–0.171)	0.143 (0.138–0.147)

Abbreviations: ACC, accuracy; ACS, acute coronary syndrome; CI, confidence interval; GRACE, global registry of acute coronary events; NPV, negative predictive value; PPV, positive predictive value.

### 3.3 Model calibration

**Figure 2:**
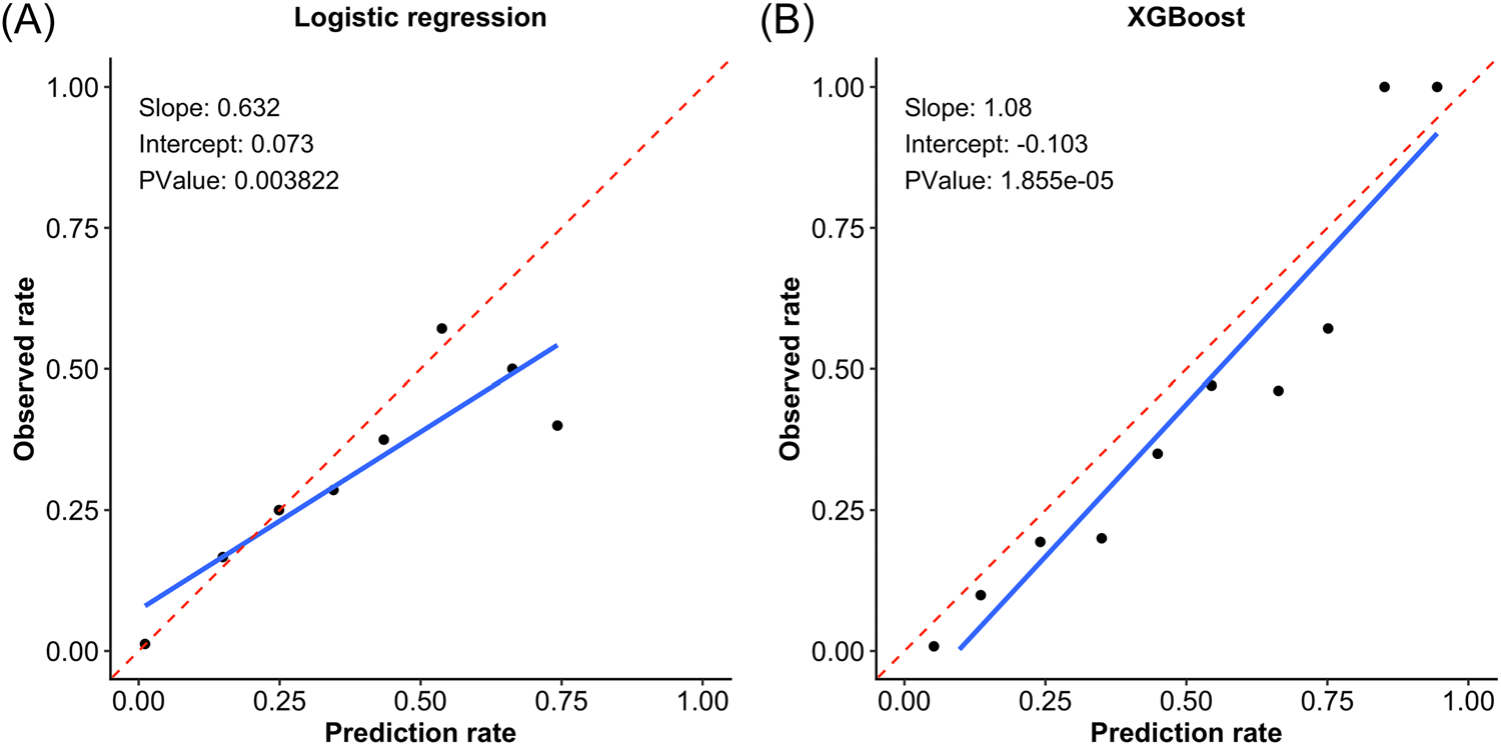
Calibration curve of logistic regression (A) and XGBoost (B) on the selected variable set

Table 2 shows the brier scores of LR and XGBoost models. Regardless of the GRACE or selected variable set, brier scores of XGBoost models were lower than LR models, respectively. Brier scores of the two models on the selected variable set were also smaller than the GRACE variable set, respectively. In addition, the calibration slope of the XGBoost model had enhanced compared to the LR model when applied to the expanded variable set (Figure 2). The slope of XGBoost model was closer to 1 and *p* value of the calibration curve was far lower than .001.

### 3.4 Model interpretation

**Figure 3:**
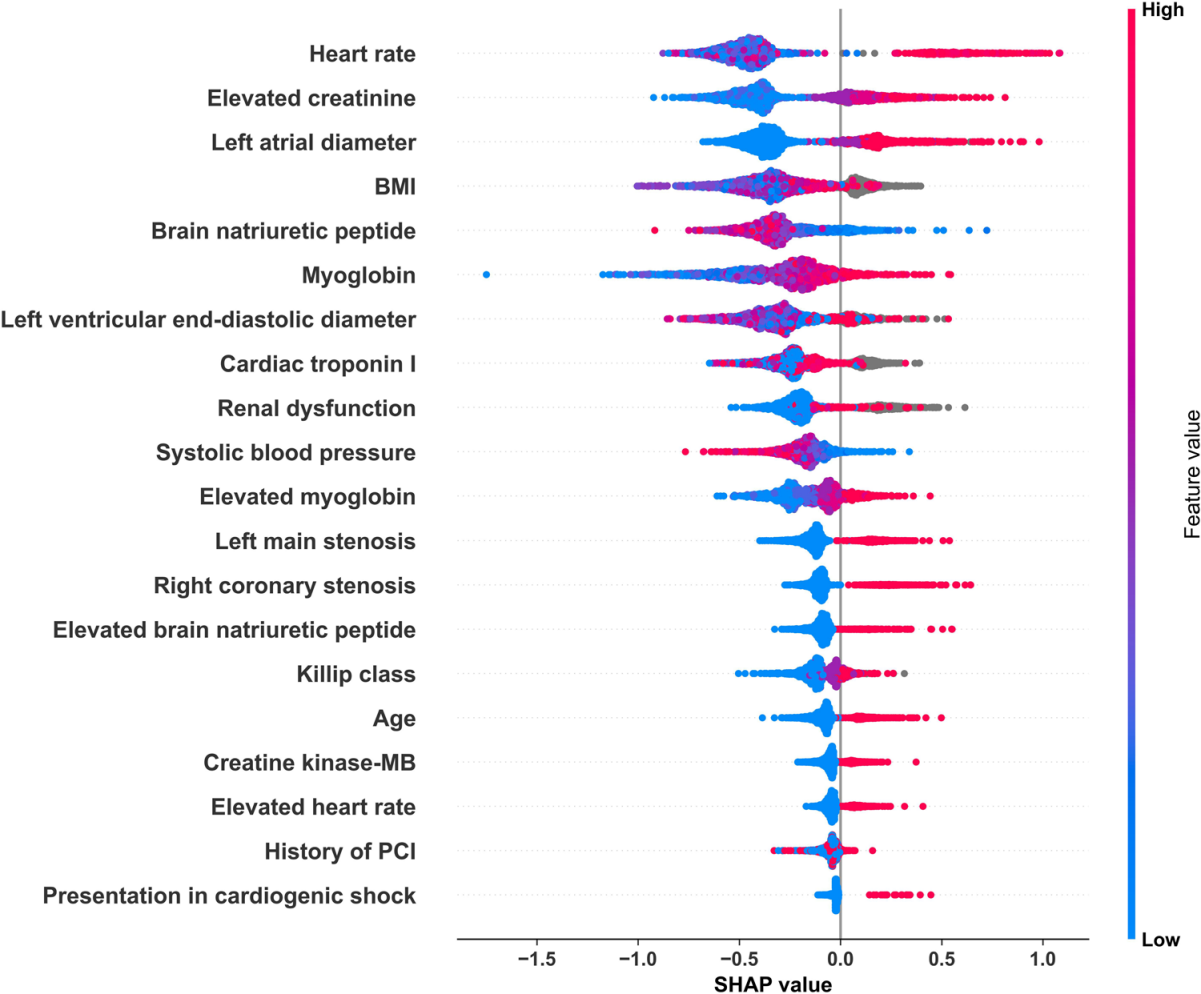
SHAP beeswarm plot* of XGBoost on the expanded variable set. *The beeswarm plot shows both variable importance and variable effects. Each point on the plot represents a sample. The color represents the value of variables. Blue means the value is low and red means high. The SHAP value (*X*‐axis) represents the impact of the variable value on model input. The SHAP value above 0 suggests increased prediction probability. And higher SHAP values suggest a higher risk of mortality (in our study). Beeswarm plot shows only the overall relationship in variable value and the prediction since the color represents only relative magnitude rather than the exact value. BMI, body mass index; MB, myoglobin; PCI, percutaneous coronary intervention; SHAP, Shapley additive explanations.

A SHAP plot demonstrates 20 significant variables ranked by importance selected by the XGBoost model on the expanded variable set (Figure 3). Generally, the risk of in‐hospital mortality rises with higher values of these continuous variables: HR, age, MB, left atrial diameter, left ventricular end‐diastolic diameter, right coronary stenosis, BNP, left main stenosis, CK‐MB, cTnI, and Killip class. Clinical events associated with these variables also contribute to the occurrence of in‐hospital death outcomes: renal dysfunction, elevated Cre, elevated MB, history of PCI, presentation in cardiogenic shock, elevated BNP, and elevated HR. In addition, higher body mass index (BMI) and SBP are protective factors of in‐hospital mortality.

Since the color represents only relative magnitude rather than the exact value, the beeswarm plot shows only the overall relationship between variable values and the prediction. To see the exact form of the relationship, SHAP dependence plots were shown in Supporting Information: Figure S2. SHAP values increased with age. For most patients under 60 years old, SHAP values were lower than −1, which means they had much lower mortality risks than the average. For most patients above 90 years old, SHAP values tended to cross the zero line, which means they had higher mortality risks. Almost all SHAP values of patients with MB above 70 µmol/L were higher than 0. There was a U‐turn on the dependence plot of the left ventricular end‐diastolic diameter. Similar U‐turns also exist on plots of HR, BMI, and left atrial diameter. The U‐turn indicates that both low and high values would contribute to a higher risk of mortality. In addition, patients admitted with cardiogenic shock had much higher SHAP values than those without. ST‐segment‐related variables were not the 20 most important variables. However, there was a clear separation between the SHAP values of different states.

## 4 DISCUSSION

In our study, a combination of 44 candidate variables was derived from 67 original variables after conducting a series of univariate and multivariate stepwise regression analyses. Furthermore, XGBoost picked out 20 vital variables from the pool of 44 variables and ranked them by their contribution to the outcome prediction without prior clinical knowledge. They demonstrated better capability for predicting outcomes than eight independent risk factors that came from the GRACE score (Table 2).

In this study, the top 20 ranked features included HR, age, SBP, Killip class, and previous PCI which was consistent with the prior studies.^2^, ^3^, ^4^, ^5^, ^6^, ^7^, ^22^ As is shown in Supporting Information: Figure S2, as the age increased, the SHAP value also increased. This indicated that advanced age is a major risk factor for in‐hospital death. However, many patients over 80 years of age had SHAP values lower than 0, which indicated that many old patients survived after emergency treatment. This suggests a trend in which the age‐related risk may be alleviated as a consequence of the improvement of PCI technologies and the speed of emergency procedures, especially in high‐level hospitals. Besides, cardiac markers (TnI, CK‐MB) and serum creatine have been demonstrated to be associated with adverse outcomes.^2^, ^5^, ^23^, ^24^, ^25^ Our findings are consistent with those studies mentioned above. However, the availability, accuracy, and reference values of TnI, MB, or CK‐MB vary greatly due to the different detection techniques in various centers. Attention should be paid to the correction of these values when processing data from multicenter studies. The last significant risk factor is cardiogenic shock, which is consistent with previous studies.^5^, ^6^, ^7^, ^26^ The total number of patients admitted with cardiogenic shock was less than 1% in our dataset. SHAP calculated the global importance of variables, which means it adds SHAP values to all samples. Therefore, the importance would be weakened if the variable only influence a few samples. This is why cardiogenic shock was not top‐ranked on the beeswarm plot. Cardiogenic shock is still an important risk factor for patients admitted with it according to its dependence plot (Supporting Information: Figure S2). Coronary stenosis was a consequential predictor in our model. The likelihood of in‐hospital death increases with coronary stenosis (left main and right coronary artery).

There were some novel findings in our study. BMI is an important predictor of in‐hospital mortality and the risk increases with lower BMI in our model. This is a phenomenon known as the obesity paradox, which has been carried out by many studies.^27^ Several mechanisms to explain the obesity paradox have been proposed. First, complex lesions as assessed on SYNTAX score and multivessel coronary artery disease were less common in obese patients.^28^, ^29^ In addition, overweight patients may have had enough nutritional reserves to sustain organ function when metabolic demands increased rapidly.^30^ Finally, obese patients were more likely to receive optimal therapy^31^ like medical therapy or invasive coronary intervention.

Variables associated with cardiac structure play important roles in our model. Left atrial diameter and left ventricular end‐diastolic diameter are both used to assess cardiac function and are associated with cardiac remodeling due to long‐term chronic diseases, such as hypertension and coronary artery diseases.

BNP, a key indicator in the diagnosis of heart failure, is also mentioned in the ranking list of SHAP. It is believed that serious myocardial infarction long‐time interruption of blood supply to the myocardium and irreversible heart damage. The larger the infarct size is, the higher the incidence of heart failure will be. The results of our model indicate that the incidence of heart failure or structural changes in the heart may also affect the severity and mortality of ACS.

The purpose of this study is not to replace the existing model, but to improve the prediction accuracy as much as possible based on the existing models. Although our model incorporates more variables than existing models, with the improvement of health informatization and the construction of specific disease databases, predict information can be obtained and prediction results can be generated in real‐time to assist doctors in clinical practice. In addition, the results of this study also found some new predictors, providing some new perspectives for updating existing models.

Moreover, a novel ML technique called XGBoost modeling was applied to evaluate the risk of in‐hospital death for patients with ACS. This advanced method allows us to develop and validate a better‐performing predictive model compared to the conventional LR technique.^32^ The algorithm of XGBoost is a highly efficient, scalable ensemble of the classification and regression tree methods with the feature of insensitivity to missing values which is more suitable for clinical use due to a large number of missing values in clinical practice. This algorithm may perform better than other ML techniques in clinical prediction because of its advantage in the processing of complex and nonlinear patterns. For example, it could identify the predictors of volume‐responsive acute kidney injury (VR‐AKI) that were not apparent using LR, resulting in a better‐performing predictive model to identify patients with VR‐AKI.^33^ Analogously, XGBoost has been used for predicting in‐hospital mortality in a large cohort of patients undergoing PCI across New York State between 2004 and 2012 previously.^34^ In a retrospective analysis of 195 patients with acute ischemic stroke, XGBoost demonstrated significantly better performance in infarct prediction compared with a standard generalized linear model in both CV approaches.^35^ Also, XGBoost could be combined with recursive feature elimination to establish an ML prognostic evaluation model for young patients with hypertension.^36^ As shown in Table 2 and Figure 1, XGboost performed better discrimination than the traditional LR method for in‐hospital mortality in patients with ACS. Probability calibration curves also implied that XGboost has more excellent performance and accuracy than the LR model (Figure 2).

The present study has several limitations. First, external validation on a separate cohort is required before the application of predictive models. Although we performed k‐fold CV and reported the results as mean values, it would still be imperative to verify the model using another population to avoid over‐fitting. Second, the dataset of this study was collected from a retrospective review without follow‐up in a single center. Therefore, whether XGboost performs as well as in other populations or holds value for long‐term prognosis is unknown.

## 5 CONCLUSION

In summary, this study developed and validated models for predicting in‐hospital mortality in a cohort of patients diagnosed with ACS in Shanghai Chest Hospital between 2015 and 2021 using XGBoost and LR. It is found that XGBoost modeling, an advanced ML algorithm, identifies new variables and provides better accuracy than LR for the prediction of in‐hospital mortality in ACS patients. These findings could highlight the utility of using novel ML approaches for the development of more precise and generalizable risk assessments in the medical field.

## AUTHOR CONTRIBUTIONS

Changqing Pan obtained research funding. Changqing Pan and Junyi Yuan conceived the study, designed the trial, and administrated and supervised the study. Rong Li and Lan Shen supervised the conduct of the study and data collection. Wenyan Ma, Bo Yan, Linfeng Li, Wenchang Chen, and Jie Zhu performed data collection. All authors analyzed the data, interpreted the result, and prepared the original draft. Changqing Pan, Junyi Yuan, Rong Li, Lan Shen, Linfeng Li, and Wenchang Chen reviewed and edited the manuscript for important intellectual content and provided administrative, medical, or technical support.

## CONFLICT OF INTEREST

The authors declare no conflict of interest.

## Supporting information

Supporting information.

## Data Availability

The data that has been used is confidential.
