# Supplementary material for: Use of machine learning models to predict in‐hospital mortality in patients with acute coronary syndrome
Source: Clin Cardiol. 2022 Dec 7;46(2):184–94. doi: 10.1002/clc.23957 (PMC9933107; doi:10.1002/clc.23957)

**Supplementary Content**

Table S1. Results of univariate logistic regression analyses

Table S2. Variables and absolute values of Pearson correlation coefficient (|PCC|) for variables whose |PCC|>0.6

Figure S1. Heatmap of |PCC| for variables whose |PCC|>0.6

Table S3. Variables of the GRACE, candidate and selected set

Table S4. Missing rates of variables

Figure S2. Dependence plots of top 20 important variables

**Table S1 Results of univariate logistic regression analyses**

| **Characteristic** | **OR(95%CI)** | **P value** |
| --- | --- | --- |
| Demographic characteristic |  |  |
| Male sex | 0.59 (0.37, 0.93)** | 0.023 |
| Age, per 10-year increse | 2.01 (1.63, 2.47) | 0 |
| BMI, per 5-kg/m^2^ increase | 0.44 (0.31, 0.62) | 0 |
| Marital status |  |  |
| Married (reference group) | 1 |  |
| Unmarried | 4.61 (0.55, 38.71) | 0.159 |
| Other | 0.0 (0.0, inf) | 0.999 |
| Type of insurance |  |  |
| Type 1* | 2.79 (1.59, 4.91) | 0 |
| Type 2* (reference group) | 1 |  |
| No insurance | 0.35 (0.19, 0.63)** | 0 |
| Other | 0.0 (0.0, inf) | 0.999 |
| Medical and personal history |  |  |
| DM | 1.99 (1.29, 3.06) | 0.002 |
| HTN | 0.86 (0.55, 1.35) | 0.512 |
| CVA | 1.39 (0.74, 2.59) | 0.306 |
| HF | 1.32 (0.63, 2.78) | 0.466 |
| MI | 1.49 (0.35, 6.3) | 0.586 |
| PAD | 1.46 (0.72, 2.96) | 0.297 |
| COPD | 3.33 (1.16, 9.62) | 0.026 |
| HLP | 0.62 (0.22, 1.71) | 0.355 |
| Renal dysfunction | 6.43 (4.03, 10.24) | 0 |
| CABG | 1.06 (0.25, 4.41) | 0.941 |
| PCI | 0.78 (0.49, 1.26) | 0.315 |
| Family history of CHD | 0.0 (0.0, inf) | 0.999 |
| Smoking | 0.65 (0.36, 1.16) | 0.141 |
| Drinking | 0.68 (0.25, 1.88) | 0.455 |
| Presentation |  |  |
| HR, per 20-beats/min increase | 3.26 (2.57, 4.14) | 0 |
| SBP, per 20-mmHg increase | 0.53 (0.43, 0.66) | 0 |
| DBP, per 20-mmHg increase | 0.4 (0.28, 0.57) | 0 |
| CHF | 6.91 (4.25, 11.24) | 0 |
| CS | 30.92 (12.49, 76.52) | 0 |
| SCA | 9.23 (0.95, 89.67) | 0.055 |
| Killip, per increase in class | 2.87 (2.24, 3.68) | 0 |
| NYHA, per increase in class | 3.31 (2.11, 5.19) | 0 |
| Presentation UCG findings |  |  |
| LVEF, per 5% increase | 0.61 (0.55, 0.68) | 0 |
| LVESD, per 5-mm increase | 1.54 (1.34, 1.77) | 0 |
| LVEDD, per 5-mm increase | 1.29 (1.07, 1.57) | 0.009 |
| LAD, per 5-mm increase | 1.65 (1.3, 2.09) | 0 |
| MR, per increase in class | 2.75 (1.92, 3.94) | 0 |
| Presentation ECG findings |  |  |
| ST-segment elevation | 2.39 (1.55, 3.69) | 0 |
| ST-segment depression | 2.05 (1.16, 3.65) | 0.014 |
| ST-segment deviation | 1.83 (1.18, 2.84) | 0.007 |
| T wave Inverted | 0.56 (0.26, 1.22) | 0.145 |
| ST-T deviation | 0.8 (0.48, 1.33) | 0.389 |
| CLBBB | 5.13 (2.09, 12.56) | 0 |
| CRBBB | 3.6 (2.04, 6.36) | 0 |
| Initial laboratory values |  |  |
| Cre, per increase in dicretization class | 2.01 (1.57, 2.59) | 0 |
| cTnI, per increase in dicretization class | 1.7 (1.42, 2.03) | 0 |
| MB, per increase in dicretization class | 2.24 (1.83, 2.75) | 0 |
| CK-MB, per increase in dicretization class | 1.99 (1.49, 2.66) | 0 |
| BNP, per increase in dicretization class | 2.06 (1.75, 2.42) | 0 |
| In-hosipital status variation |  |  |
| elevated HR | 1.96 (1.2, 3.18) | 0.007 |
| elevated SBP | 1.26 (0.66, 2.4) | 0.486 |
| elevated DBP | 2.22 (1.09, 4.54) | 0.029 |
| elevated Cre | 4.75 (3.04, 7.42) | 0 |
| elevated cTnI | 0.74 (0.46, 1.19) | 0.216 |
| elevated MB | 3.11 (1.96, 4.95) | 0 |
| elevated CK-MB | 1.16 (0.7, 1.94) | 0.561 |
| elevated BNP | 1.92 (1.14, 3.24) | 0.015 |
| HR change, per beats/min increase | 1.05 (1.04, 1.07) | 0 |
| DBP change, per mmHg increase | 0.99 (0.95, 1.04) | 0.823 |
| SBP change, per mmHg increase | 1.03 (1.0, 1.05) | 0.056 |
| Cre change, per umol/L increase | 1.03 (1.02, 1.04) | 0 |
| cTnI change, per ng/mL increase | 1.04 (1.01, 1.07) | 0.009 |
| MB change, per ng/mL increase | 1.0 (1.0, 1.0) | 0 |
| CK-MB change, per ng/mL increase | 0.99 (0.99, 1.0) | 0.157 |
| BNP change, per pg/mL increase | 1.0 (1.0, 1.0) | 0.004 |
| In-hospital surgical characteristics |  |  |
| CABG | 0.63 (0.25, 1.58) | 0.329 |
| PCI | 0.4 (0.26, 0.62) | 0 |
| LM stenosis, per 10% increase | 3.27 (1.66, 6.44) | 0 |
| LAD stenosis, per 10% increase | 2.08 (0.92, 4.7) | 0.004 |
| LCX stenosis, per 10% increase | 0.93 (0.51, 1.69) | 0.718 |
| RCA stenosis, per 10% increase | 1.66 (0.88, 3.16) | 0.032 |
| Abbreviations: DM, diabetes mellitus; HTN, hypertension; CVA, cerebral stroke; HF, heart failure; MI, myocardial infarction; PAD, peripheral artery disease; COPD, chronic obstructive pulmonary disease; HLP, hyperlipidemia; CABG, coronary artery bypass graft; PCI, percutaneous coronary intervention; CHD, coronary heart disease; BMI, body mass index; HR, heart rate; SBP, systolic blood pressure; DBP, diastolic blood pressure; CHF, congestive heart failure; CS, cardiogenic shock; SCA, sudden cardiac arrest; ECG, electrocardiography; UCG, echocardiography; LVEF, left ventricular ejection fraction; LVESD, left ventricular end-systolic diameter; LVEDD, left ventricular end-diastolic diameter; LAD, left atrial diameter; MR, mitral regurgitation; CLBBB, complete left bundle branch block; CRBBB, complete right bundle branch block; Cre, creatinine; cTnI, cardiac troponin I; MB, myoglobin; CK-MB, creatine kinase-MB; BNP, brain natriuretic peptide; LM, left main; LAD, left anterior descending; LCX, left circumflex; RCA, right coronary artery.  * Type 1: Non-employed resident medical insurance; Type 2: Urban employee medical insurance  **We found that age was a confounding factor for no insurance. Patients without insurance are 7.6 years younger than patients with urban employee medical insurance (63.3 vs 70.9). OR values here were misleading. And we found that this would result in a lower model performance. Therefore, we excluded insurance during variable selection. | | |

**Table S2 variables and absolute values of Pearson correlation coefficient (|PCC|) for variables whose |PCC|>0.6**

| **Variable 1** | **Variable 2** | **|PCC|** |
| --- | --- | --- |
| PCI | CSI | 0.886 |
| LVESD | LVEDD | 0.858 |
| cTnI | CK-MB | 0.792 |
| LVEF | LVESD | 0.782 |
| ST-segment elevation | ST-segment deviation | 0.729 |
| LVEF | Presentation in CHF | 0.727 |
| MB | CK-MB | 0.694 |
| LVEF | BNP | 0.621 |
| MB | cTnI | 0.610 |
| CHF | LVESD | 0.604 |
| cTnI change | CK-MB change | 0.600 |

**Figure S1 Heatmap of |PCC| for variables whose |PCC|>0.6**


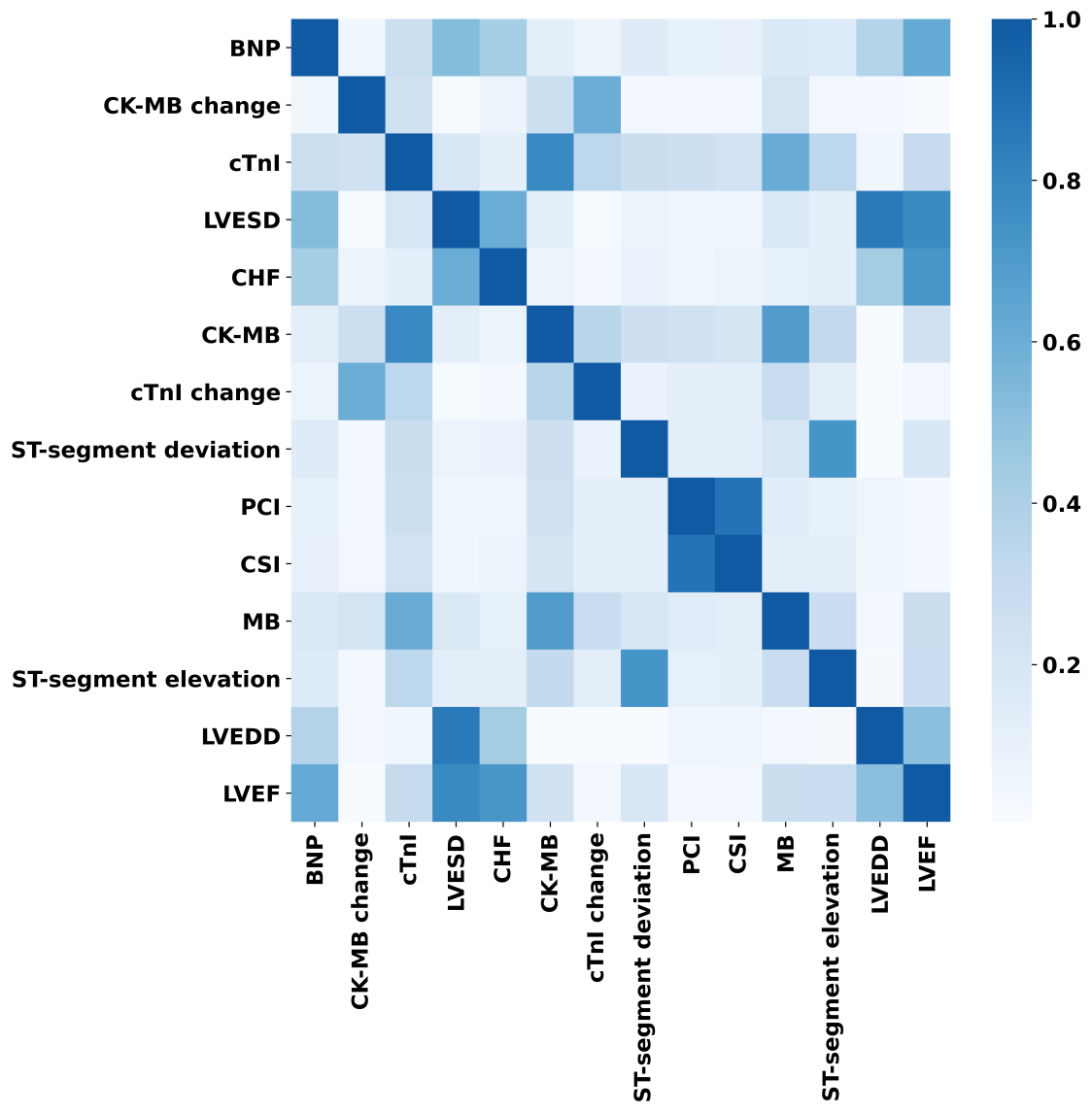


**Table S3 variables of the GRACE, candidate and selected variable set**

| **GRACE variables** | **Candidate variables for model construction** | **Selected variables for model construction** |
| --- | --- | --- |
| Killip, SBP, HR, Age, Cre, Presentation after CA, ST-segment deviation, initial cardiac markers positive | Age, Sex, BMI, HR, SBP, DM, HF, MI, COPD, Renal dysfunction, History of PCI, CHF, CS, SCA, Killip, NYHA, MR, ST-segment elevation, ST-segment depression, ST-segment deviation, CLBBB, CRBBB, LVEF, LVEDD, LAD, Cre, cTnI, CK-MB, BNP, MB, elevated HR, elevated DBP, elevated Cre, elevated MB, elevated BNP, HR change, Cre change, cTnI change, MB change, BNP change, CSI, LM stenosis, LAD stenosis, RCA stenosis | HR, elevated Cre, LAD, BMI, BNP, MB, LVEDD, cTnI, Renal dysfunction, SBP, elevated MB, LM stenosis, RCA stenosis, elevated BNP, Killip, Age, CK-MB, elevated HR, History of PCI, CS |

**Table S4 Missing rates of variables**

| **Variable** | **Missing Values** | **% of Total Values** |
| --- | --- | --- |
| LAD | 478 | 19.8 |
| RCA stenosis | 399 | 16.5 |
| LM stenosis | 395 | 16.4 |
| LAD stenosis | 394 | 16.3 |
| LVEDD | 344 | 14.3 |
| MR | 343 | 14.2 |
| LVEF | 299 | 12.4 |
| BNP | 109 | 4.5 |
| elevated BNP | 109 | 4.5 |
| BNP change | 109 | 4.5 |
| BMI | 106 | 4.4 |
| MB | 62 | 2.6 |
| elevated MB | 62 | 2.6 |
| MB change | 62 | 2.6 |
| Cre | 48 | 2 |
| elevated Cre | 48 | 2 |
| Cre change | 48 | 2 |
| cTnI | 40 | 1.7 |
| cTnI change | 40 | 1.7 |
| CK-MB | 39 | 1.6 |
| HR | 11 | 0.5 |
| elevated HR | 11 | 0.5 |
| HR change | 11 | 0.5 |
| SBP | 9 | 0.4 |
| elevated DBP | 9 | 0.4 |

**Figure S2 Dependence plots of top 20 important variables**


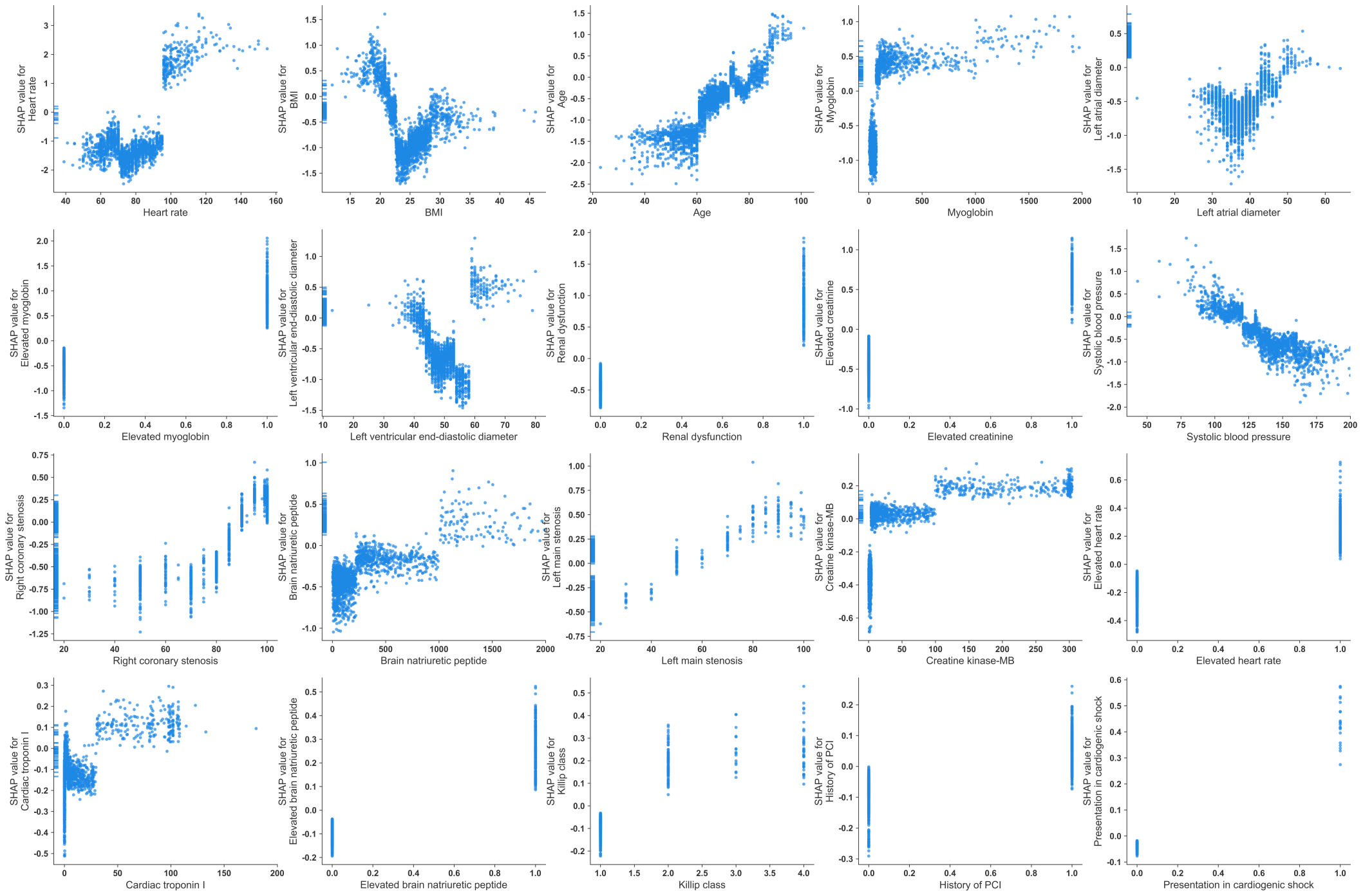

Supplement: Supplementary file 1 — Supporting information. [file CLC-46-184-s001.doc]
